# Validation of a multi-omics strategy for prioritizing personalized candidate driver genes

**DOI:** 10.18632/oncotarget.9540

**Published:** 2016-05-21

**Authors:** Li Liang, Liting Song, Yi Yang, Ling Tian, Xiaoyuan Li, Songfeng Wu, Wenxun Huang, Hong Ren, Ni Tang, Keyue Ding

**Affiliations:** ^1^ Key Laboratory of Molecular Biology for Infectious Diseases (Ministry of Education), Institute for Viral Hepatitis, Department of Infectious Diseases, The Second Affiliated Hospital, Chongqing Medical University, Chongqing, 400010 PR China; ^2^ Department of Medical Oncology, Peking Union Medical College Hospital, Peking Union Medical College and Chinese Academy of Medical Sciences, Beijing, 100730 P.R. China; ^3^ State Key Laboratory of Proteomics, National Protein Science Beijing Center, Beijing Proteome Research Center, Beijing Institute of Radiation Medicine, Beijing, 102206 P.R. China

**Keywords:** personalized mutation-driver genes, multi-omics, validation, structure-function relationship, in vitro experiment

## Abstract

Significant heterogeneity between different tumors prevents the discovery of cancer driver genes, especially in a patient-specific manner. We previously prioritized five personalized candidate mutation-driver genes in a hyper-mutated hepatocellular carcinoma patient using a multi-omics strategy. However, the roles of the prioritized driver genes and patient-specific mutations in hepatocarcinogenesis are unclear. We investigated the impact of the tumor-mutated allele on structure-function relationship of the encoded protein and assessed both loss- and gain-of-function of these genes and mutations on hepatoma cell behaviors *in vitro*. The prioritized mutation-driver genes act as tumor suppressor genes and inhibit cell proliferation and migration. In addition, the loss-of-function effect of the patient-specific mutations promoted cell proliferation and migration. Of note, the *HNF1A* S247T mutation significantly reduced the *HNF1A* transcriptional activity for hepatocyte nuclear factor 4 alpha (*HNF4A*) but did not disrupt nuclear localization of HNF1A. The results provide evidence for supporting the validity of our proposed multi-omics strategy, which supplies a new avenue for prioritizing mutation-drivers towards personalized cancer therapy.

## INTRODUCTION

Genetic alterations underlying carcinogenesis have been characterized with the advance of next-generation sequencing technology. One of the main aims of cancer-genome sequencing studies, e.g., the Cancer Genome Atlas [1] and the International Cancer Genome Consortium [2], is to identify driver genes/mutations underlying tumor initiation, maintenance, progression, and metastasis [3]. Distinguishing cancer drivers from millions of somatic mutations (e.g., passengers) remains a monumental challenge. Significantly mutated genes across multiple samples have been exploited based on the hypothesis that positive selection has operated on recurrent mutations and hence has functional relevance [4, 5]. Although TCGA identified significantly mutated genes based on mutation-frequency or -pattern, it remains difficult to prioritize specific driver genes/mutations in patient-specific manners due to substantial tumor heterogeneity. The identification of patient-specific drivers may serve as candidate drug targets for personalized therapy, as advocated in the precision medicine initiative [6].

It is of vital therapeutic potential in finding mutated peptides, which will provide a significant implication in identifying possible driver mutations [7]. In our previous study, we prioritized five personalized candidate mutation-drivers in a hyper-mutated hepatocellular carcinoma patient, which were patient-specific and not reported, by characterizing the expression of tumor-mutated alleles from genome to mRNA to protein [8]. Our proposed ‘multi-omics’ strategy utilized the mutation profile (genome, transcriptome and proteome) from a given patient rather than a population-based study, combined with the catalogue of known cancer drivers and current cancer knowledgebase. However, the roles of the prioritized driver genes and mutations in hepatocarcinogenesis are unclear.

In the present study, we aimed to provide evidence to support the validity of the multi-omics strategy by investigating the impact of missense mutations on structure-function relationship of these genes and assessing the functional effects of the prioritized genes, especially the patient-specific mutations, on cell behaviors in hepatoma cell lines.

## RESULTS

### A multi-omics strategy for prioritizing patient-specific mutation-driver genes in hepatocellular carcinoma

**Figure 1:**
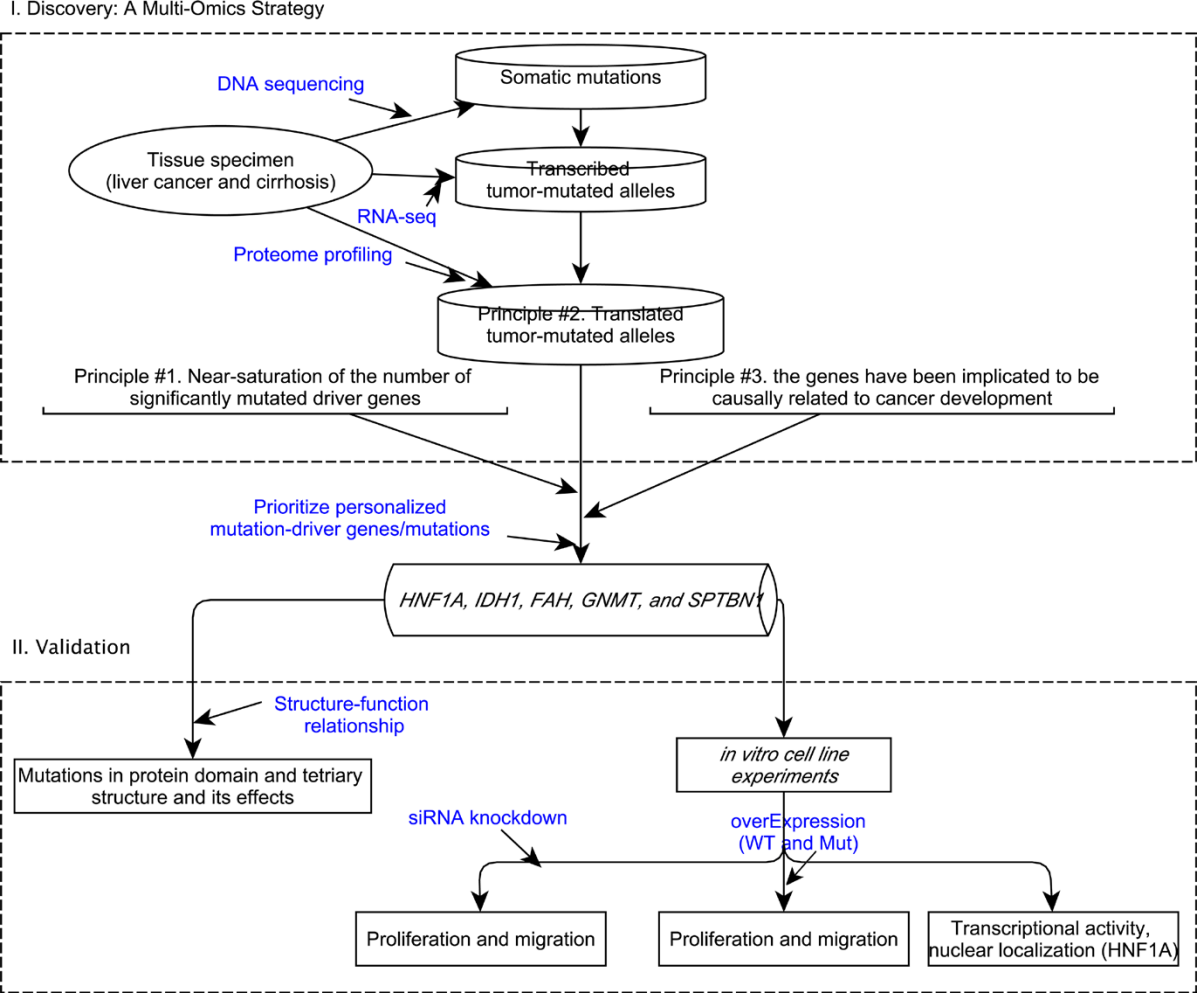
A conceptual framework to prioritize personalized candidate mutation-driver genes based on a multi-omics strategy and its validation

In our previous study [8], we employed a multi-omics strategy for prioritizing personalized candidate mutation-driver genes (Figure 1). The strategy built upon the following principles: 1) the near-saturation of the number of significantly mutated cancer drivers [9, 10]. Vogelstein [9] commented that ‘the number of frequently altered Mut-driver genes (mountains) is nearing saturation’. Lawrence et al. [10] estimated that near-saturation of significantly mutated genes may be achieved with 600-5000 samples per tumour type. The near-saturation of significant mutated genes provided a basis for prioritizing personalized mutation-driver genes; 2) the effect of tumor-mutated alleles as inferred by its expression at the mRNA and/or protein levels. A fundamental question in proteogenomics is which protein coding alterations are expressed at the protein level [7, 11]. The mutated cancer genomes produce mutant transcriptome and proteome, therefore, mutant proteins that give the cancer cell its oncogenic properties. The mutant proteins are produced only by tumor cells and have found to be functionally related to cancer driver mutations. 3) the current knowledgebase of cancer genes. A causal network analysis based on the Ingenuity Knowledge Base (IPA^®)^ [12] can be used to characterize causal effect between the genes and diseases, e.g., hepatocellular carcinoma.

We obtained high throughput sequencing data by whole-exome sequencing, transcriptomic sequencing (RNA-seq) and proteome profiling in a hyper-mutated (due to *MSH2* inactivation) hepatocellular carcinoma patient. We characterized the expression patterns of 4980 tumor-mutated alleles and found that only 42% and 3.5% tumor-mutated alleles were transcribed and translated, respectively. The screening steps in prioritizing personalized mutation-driver genes were illustated in detail previously [8]. Notably, the two most frequently mutated (~20%) genes in HCC (i.e., *TP53* and *CTNNB1*) were not mutated in this patient, and the tumor-mutation allele of a missense mutation in *ARID1A* was not transcribed. We prioritized five personalized candidate driver genes/mutations in this patient (Table 1), which were patient-specific and were not reported in hepatocellular carcinoma in the Catalog of Somatic Mutation in Cancer database (COSMIC, version 75) [13].

**Table 1:** Prioritized personalized candidate mutation-driver genes

Chr_pos	Symbol	Protein	AA_change	PDB
chr12:121431992	*HNF1A*	NP_000536.5	p.S247T	1IC8
chr6:42928545	*GNMT*	NP_061833.1	p.A14T	1R74
chr2:209104698	*IDH1*	NP_005887.2	p.V294M	1T0L
chr15:80473495	*FAH*	NP_000128.1	p.I392V	NA
chr2:54882240	*SPTBN1*	NP_003119.2	p.N1952K	NA

### Validation of the expression of mutated protein in hepatoma cell lines

Cancer cell lines are commonly used as *in vitro* models for clinical tumors although cultured cancer cells might have different genetic characteristics from *in situ* tumors [14]. We assessed both the loss- and gain-of-function of the prioritized genes (Table 1) on hepatoma cell behavior in four hepatoma cell lines (Huh7, SK-Hep1, PLC/PRF/5 and SMMC-7721), which were previously established from human primary hepatocellular carcinoma.

We first assessed the genetic aberration background of the five genes by sequencing the targeted mutation sites in hepatoma cell lines, and we did not note any changes in the sequenced nucleotide (data not shown). The expression of four targeted genes in five hepatoma cell lines and a normal hepatic cell line (LO2) were evaluated, indicating the protein expression of the targeted genes among the hepatoma and normal hepatic cell line were similar (Figure S1).

**Figure 2:**
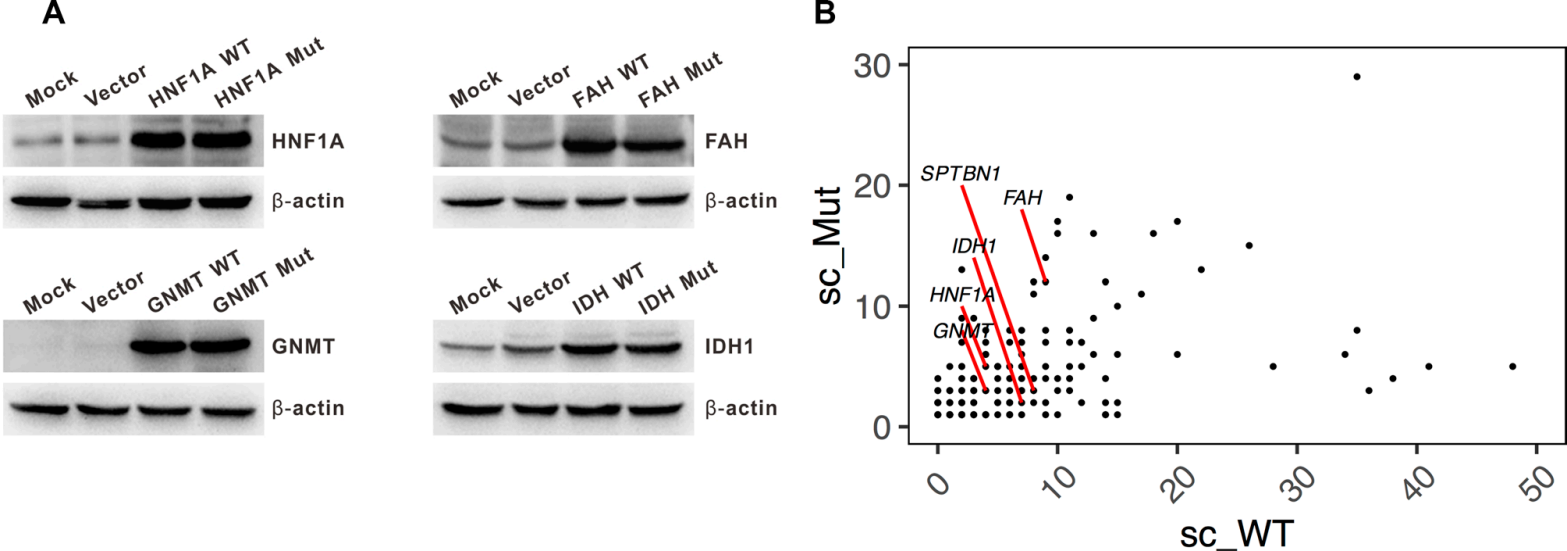
Expression of the wild-type and mutant allele of personalized candidate mutation-driver genes (**A**) Western blot showing a similar expression of the wild-type and mutant allele of *HNF1A*, *IDH1*, *GNMT*, and *FAH*. (**B**) The spectral count of wild-type (sc_WT) and mutated- (sc_Mut) amino acid identified by proteomic profiling. Spectral count (sc) is defined as the total number of spectra identified for a protein in quantitative proteomics. The sc of the five genes are shown.

We used the knockdown approach of multiple siRNAs to reduce the endogenous protein expression in hepatoma cells (Figure S2). We then constructed recombinant vectors exogenously expressing wild-type and mutant allele of *HNF1A*, *GNMT*, *IDH1* and *FAH* (*SPTBN1* was excluded due to its long coding region (7092 bp)). The expression of both the wild-type and mutant allele of these genes was similar (Figure 2A). These results confirmed our previous findings that both the wild-type and mutant allele of amino acid were identified in the liver cancer tissue by mass spectrometry [8]. A positive correlation of the spectral count of the wild-type and mutant allele of the amino acid (*r* = 0.31, *p* = 4.2 × 10^−5) from proteomics profiling was noted (Figure 2B).^

### *HNF1A* S247T mutation is a loss-of-function oncogenic event

**Figure 3:**
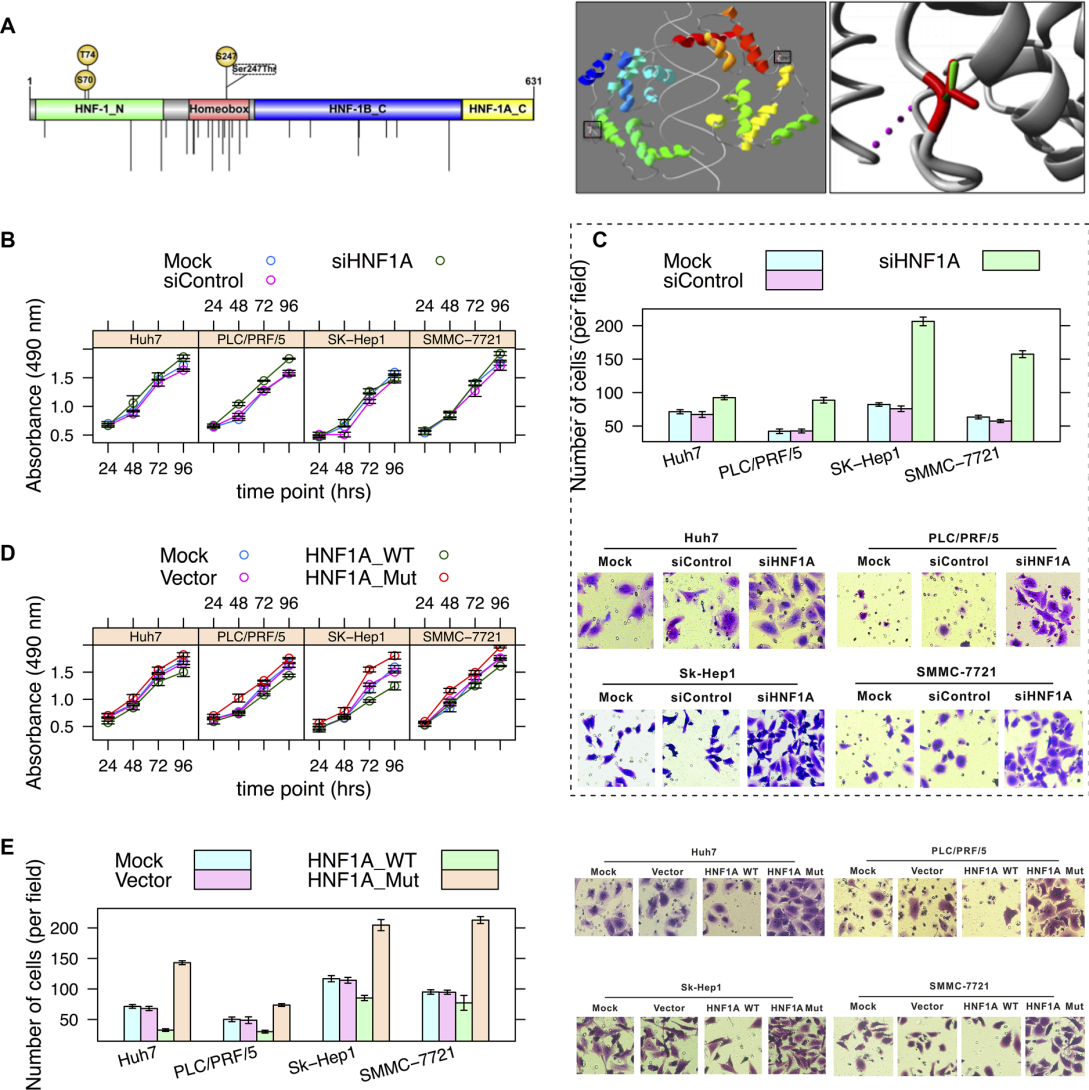
Effects of a loss-of-function mutation (S247T) in *HNF1A* (**A**) Schematic diagram of domains (left panel) and image of crystallographic model (right panel) (PDB: 1IC8) of *HNF1A*. The Ser247 (phosphoserine) residue is locates on the homeobox domain. Nonsynonymous mutations occurring in hepatocellular carcinoma (from the COSMIC database) are shown by vertical lines. The side chains of both the wild-type and mutant allele of residue are shown in green and red, respectively. (**B**) Hepatoma cell lines SMMC-7721, SK-Hep1, PLC/PRF/5 and Huh7 cells were infected with *HNF1A*-targeting siRNA (AdsiHNF1A) or nontargeting control siRNA (AdsiControl). At 12 h after infection, cells were plated into a 24-well plate at 0.5 × 10^4^/ml and were counted every 24 h in triplicate. Data are presented as the mean ± sd. (**C**) The Transwell assay of cell migration property in hepatoma cells. Cells were treated as described in Figure 3B. Quantitative evaluation of cell migration activity is presented as the means ± sd of five randomly selected microscopic fields from three independent experiments. Magnification:×200. (**D**) Hepatoma cells were mock-transfected or transfected with *HNF1A*-WT or *HNF1A*-Mut (S247T), respectively. Tumor cell growth was measured similar to Figure 3B. (**E**) Cells were treated as described in Figure 3D. Cell migration was determined by Transwell assay.

*HNF1A* is a transcription factor that is highly expressed in the liver, which is required for the regulation of the expression of several liver-specific genes, including *FGA*, *FGB*, *SERPINA1*, and *AFP* [15]. It has been suggested that bi-allelic inactivation of *HNF1A* may be an early step in the development of some hepatocellular carcinomas [16]. Somatic mutations in *HNF1A* in hepatocellular carcinoma from the COSMIC database [13] indicated that the number of non-silent mutations in the homeobox domain is significantly greater than other domains (*p* = 8.64 × 10^−6^) (Figure 3A).

We first investigated the impact of S247T on the structure-function relationship of HNF1A based on its crystallographic structure of 83-279 amino acids (PDB: 1IC8) [17] (Figure 3A). The residue is located in the surface of HNF1A, which is involved in a multimer contact according to the PISA-database [18]. The mutation introduces a larger residue (threonine) at this position and this can disturb the multimeric interactions.

Second, we evaluated the effects of knockdown of *HNF1A* on hepatoma cell growth and migration. We noted that, in comparison with siRNA control, siRNA-mediated depletion of *HNF1A* significantly promoted hepatoma cell proliferation (ANOVA, *p* = 0.001, 1.4 × 10^−6^, 0.091, and 0.025 in Huh7, PLC/PRF/5, SK-Hep1, and SMMC-7721, respectively) (Figure 3B), as well as its migratory capacity (*p* = 1.2 × 10^−5^, 1.7 × 10^−7^, 3.1 × 10^−9^, and 5.5 × 10^−8^) (Figure 3C). These results indicated that *HNF1A* loss-of-function may play an important role in hepatocellular carcinoma tumorigenecity and metastasis.

Third, we tested the effects of overexpression of *HNF1A* on hepatoma cell growth. Compared with the vector control, overexpression of wild-type *HNF1A* led to a significantly lower proliferation rate (*p* = 0.0015, 5.7 × 10^−5^, 0.0012, and 5 × 10^−6^) (Figure 3D); the migratory capacity of multiple hepatoma cells was significantly suppressed by exogenously expression of wild-type *HNF1A* (*p* = 5.5 × 10^−10^, 1.6 × 10^−4^, 1.1 × 10^−6^, and 5.5 × 10^−9^) (Figure 3E). When compared with the wild-type *HNF1A*, overexpression of the mutant allele of *HNF1A* (S247T) showed a sharply increased cell proliferation (*p* = 4 × 10^−6^, 1.4 × 10^−8^, 2.5 × 10^−5^, and 7.5 × 10^−8^) (Figure 3D) and enhanced migratory ability (*p* = 4.2 × 10^−11^, 1.5 × 10^−9^, 3.4 × 10^−7^, and 8.7 × 10^−7^) (Figure 3E). Although the protein expression of mutants was clearly detected in hepatoma cells (Figure 2A), functional studies demonstrated the opposite effects of wild-type and mutant allele of HNF1A on hepatoma cell growth and migration.

**Figure 4:**
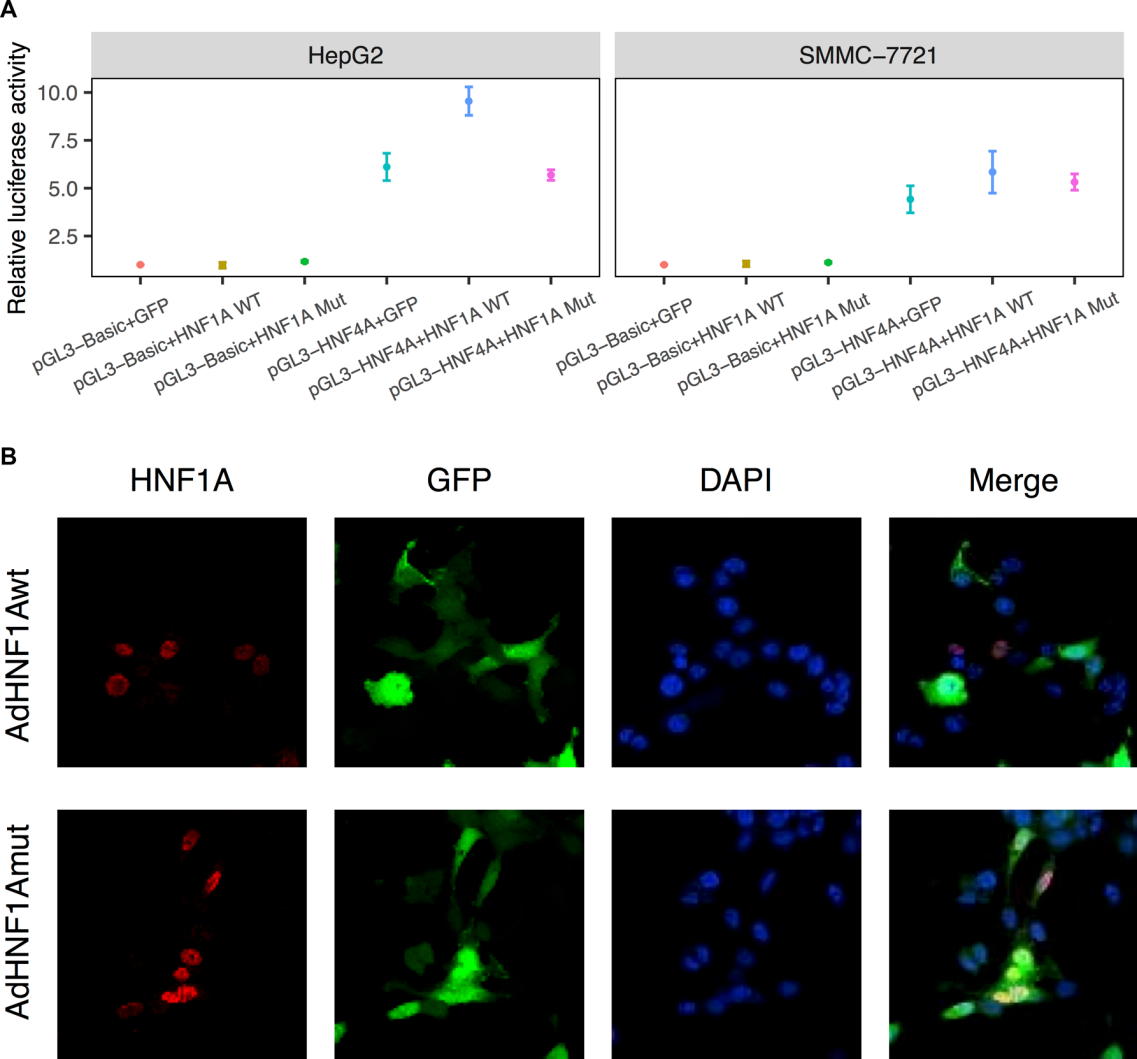
S247T mutation reduced HNF1A transactivity (**A**) Luciferase assay of human HNF4A promoter constructs in HepG2 and SMMC-7721 cells. HepG2 and SMMC-7721 cells were co-transfected with pGL3-HNF4A and pAdTrack-HNF1A (HNF1A WT) or pAdTrack-HNF1AS247T (HNF1A Mut). At 36 hours after infection, cells were collected for luciferase assays. Results are presented as the mean relative luciferase activity against the activity of the pGL3-Basic control sample ± SD of three independent experiments. (**B**) SMMC-7721 cells were infected with adenoviruses expressing wild-type (AdHNF1Awt) or mutated-HNF1A (AdHNF1Amut). Thirty-six hours after adenovirus infection, HNF1A expression was detected by immunofluorescence with an anti-HNF1A antibody and an Alexa Fluor 647-labeled secondary antibody. Cells were counterstained with DAPI to label nuclei. The cellular localization of HNF1A were visualized under a fluorescence microscope. Magnification: 600×.

Mutations in HNF1A may alter protein functions through decreased DNA binding capacity, reduced transactivation, or disrupted nuclear localization [19]. HNF1A was shown to bind to the *HNF4A* promoter region in mouse [20], and mutation in *HNF1A* demonstrated reduced transactivation activity of its target (*HNF4A*) promoter [21], which is essential for the differentiation of the hepatic lineage [22] and loss of HNF4A is a critical event in the progression of hepatocellular carcinoma [23]. Therefore, we first examined the functional consequences of the S247T mutation on *HNF1A* transcriptional activity by using the *HNF4A* promoter reporter (Figure 4A). The results demonstrated that the wild-type HNF1A resulted in an enhancement of transcriptional activity of pGL3-HNF4A in HepG2 and SMMC-7721 cells (*t*-test, *p* = 0.004 and 0.044, respectively). Compared with the wild-type HNF1A, the reporter activity of S247T mutation was reduced by 40% and 9% (*p* = 0.006 and 0.058). Next, we examined the cellular distribution of HNF1A in hepatoma cells transduced with wild-type HNF1A as well as S247T-mutated HNF1A (Figure 4B). The nuclear localization of cells infected with wild-type or mutated AdHNF1A did not differ in SMMC-7721 cells. These results indicated that *HNF1A* has a tumor suppressive effect and a loss-of-function mutation (S247T) in the homeobox domain leads to an oncogenic effect by affecting transcriptional activity.

### The V294M mutation in *IDH1*: loss-of- or gain-of-function?

**Figure 5:**
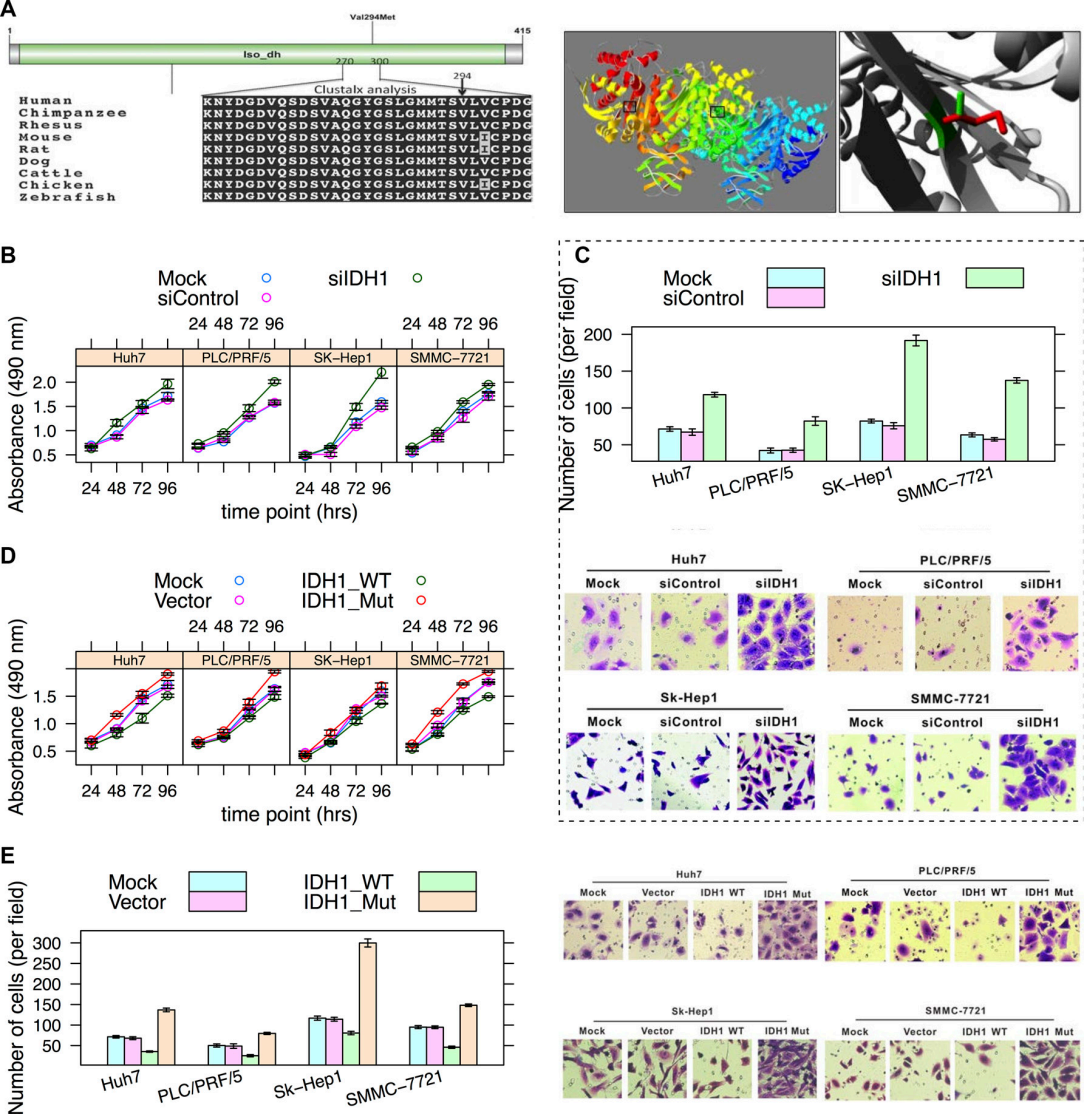
Effects of V294M in *IDH1* (**A**) Schematic diagram of domains and multiple sequence alignment of IDH1 (270-300 AAs) (left) and image of a crystallographic model (right) (PDB: 1T0L) of *IDH1* [49]. The V294 residue resides in the iso-dh domain. The side chains of both the wild-type and mutant allele of the residue are shown in green and red, respectively (right). (**B**) Cell growth curve. Hepatoma cell lines SMMC-7721, SK-Hep1, PLC/PRF/5 and Huh7 cells were infected with AdsiIDH1 or AdsiControl, respectively. Cell growth was determined by MTS assay similar to Figure 3B. (**C**) The Transwell assay of cell migration property in hepatoma cells depletion of IDH1. Magnification:×200. (**D**) Hepatoma cells were mock-transfected or transfected with *IDH1*-WT and *IDH1*-Mut (V294M), respectively. Tumor cell growth was measured by MTS assay. (**E**) Cells were treated as described in Figure 3D. Cell migration was determined by Transwell assay.

Isocitrate dehydrogenases (e.g., IDH1) catalyze the oxidative decarboxylation of isocitrate to 2-oxoglutarate. Mutations in *IDH1* have been reported in many types of tumors, e.g., gliomas, acute myeloid leukemias, and intrahepatic cholangiocarcinomas [24]. A high mutation frequency of the R132 residue was noted in gliomas and acute myeloid leukemias. Evidence suggested that the *IDH1* mutation may be an early event in tumorigenesis with multiple downstream oncogenic consequences [25]. The residue (V294) is located in the isopropylmalate dehydrogenase-like (iso-dh) domain, which is highly conserved across homologous sequences and the mutated-allele was not observed (Figure 5A, left panel). This domain is important for the activity of the protein and is in contact with residues. The mutation (V294M) can affect this interaction and thus protein function (Figure 5A, right panel).

Whether *IDH1* is an oncogene or a tumor suppressor gene is still debated [26]. Our *in vitro* cell experiment suggested that *IDH1* may act as a tumor suppressor gene in hepatocellular carcinoma in that loss-of-function of *IDH1* significantly promoted hepatoma cell growth (Figure 5B) and migration (Figure 5C); whereas overexpression of wild-type IDH1 had opposite effects (Figure 5D–5E). In contrast, the V294M mutation in *IDH1* resulted in the promotion of cell growth and migration (Figure 5D–5E), suggesting a loss-of-function effect of V294M.

### GNMT, *FAH* and *SPTBN1*

A *Gnmt* knockout mouse model of hepatocellular carcinoma has shown that genes related to the Wnt pathway (e.g., *Ctnnb1*, *Ccnd1*, and *Myc*) were up-regulated [27], indicating that *GNMT* is a tumor suppressor gene for liver cancer. GNMT is an enzyme that catalyzes the conversion of S-adenosyl-L-methionine (SAM) to S-adenosyl-L-homocysteine and sarcosine, which is involved in many essential cellular processes including biosynthesis, signal transduction, protein repair, chromatin regulation and gene silencing [28]. The residue (A14) is part of an Interpro domain of SAM-dependent methyltransferase (Figure S3A, left panel), which is important for the activity of the protein and in contact with other residues. The hydrophobicity of the wild-type (Alanine) and mutant (Threonine) residue differs, which may cause loss of hydrophobic interactions with other molecules on the surface of the protein. We noted that loss-of-function of *GNMT* by siRNA promoted hepatoma cell growth (Figure S3B) and migration (Figure S3C) in comparison with siRNA control. In addition, overexpression of the wild-type and mutant allele of *GNMT* had opposite effects (Figure S3D–S3E), i.e., inhibition or promotion of cell proliferation and migration, respectively.

*FAH* was mainly expressed in the liver and kidney [29, 30], which is the last enzyme in the tyrosine catabolism pathway that synthesizes acetoacetate and fumarate from L-phenylalanine [31, 32]. A deficiency of *FAH* is associated with type 1 hereditary tyrosinemia [33, 34]. The exact crystal-structure of FAH is unknown, and we performed homologous modeling of *FAH* upon mouse Fah (PDB: 1HYO) (Figure S4A). The residue (I392) is located in the FAA hydrolase domain which functions in hydrolase and catalytic activities. The mutant residue (Valine) is smaller than the wild-type (Isoleucine) residue, which will cause an empty space in the protein core. Therefore, the mutation may affect the domain interaction and protein function. We found that loss-of-function of *FAH* promoted hepatoma cell growth (Figure S4B) and migration (Figure S4C) in comparison with siRNA controls. Additionally, overexpression of the wild-type and mutant allele of *FAH* had opposite effects (Figure S4D–S4E), i.e., inhibiting or promoting cell proliferation and migration, respectively.

*SPTBN1* plays an important role in the determination of cell shape, the arrangement of transmembrane proteins and the organization of organelles [35]. A recent report showed that by regulating the Wnt inhibitor kallistatin, loss of *SPTBN1* activates Wnt signaling and promotes progression of hepatocellular carcinoma and Wnt signaling [36]. The mutation (N1952K) is located in one of the spectrin repeats (Figure S5A). We constructed the protein structure of *SPTBN1* by homologous modeling using SWISS-MODEL [37] but with a GMQE (Global Model Quality Estimation) of 0.16, indicating a lower reliability. Loss-of-function of *SPTBN1* promoted hepatoma cell proliferation and migration (Figures S5B–S3D).

## DISCUSSION

In the present study, we provided supporting evidence for validating the patient-specific mutation-driver genes that was prioritized based on a multi-omics strategy (Figure 1) [8]. We hypothesized that the translated tumor-mutated allele would impact the structure-function relationship of the encoded protein. Therefore, the mutant-type of protein may play an important role in carcinogenesis.

Our results suggested that the prioritized mutation-driver genes act as tumor suppressor genes instead of oncogenes to regulate cell cycle. The *in vitro* evidence showed that loss- and gain-of-function of these genes had obvious effects on cell proliferation and migration in four hepatoma cell lines. In addition, we confirmed that the mutant-type protein was expressed *in vitro* (Figure 2), and the patient-specific missense mutations in four genes have similar loss-of-function effects. Of note, we characterized the functional consequence of S247T in *HNF1A*, in which phosphorylation of HNF1A at Ser247 was involved in *HNF1A* transcriptional activity [38]. Although both the wild- (serine) and mutant- (threonine) type can be phosphorylated, significantly reduced transcriptional activity was noted in the HNF1A mutant-type for HNF4A reporter (Figure 4A).

*IDH1* was thought to be an oncogene whose mutations have stimulated the burgeoning field of tumor metabolism [9]. There is a mutation hotspot (R132H) in *IDH1* in glioma [39] and acute myeloid leukemia [40] that is responsible for driving tumor progression. However, the hotspot R132H mutation was significantly under-represented in intrahepatic cholangiocarcinoma [41], suggesting a differential mutation pattern of IDH1 in different cancer types. Mutant *IDH1* inhibits HNF4A to block hepatocyte differentiation and promote intrahepatic cholangiocarcinomas [42]. The promotion of cell proliferation and migration by siRNA knockdown (Figure 5B–5C), as well as its effects by overexpression of the wild-type and mutant allele of IDH1 (Figure 5D–5E), indicated that *IDH1* may act as a tumor suppressor gene on hepatoma cell lines.

There are several limitations in the present study. First, we adapted tumor cells as model, which may not support the key role of driver genes in tumor cell transformation. However, the prioritized genes/mutations modulate tumor cell growth and aggressiveness (i.e., progression), which features one of malignant characteristics of tumor cells. Second, it is unclear whether there are synergistic effects of these candidate driver genes.

In conclusion, we validated the strategy for prioritizing personalized mutation-driver genes using multi-omics data. Our study provided evidences that these expressed mutations exert potential oncogenic effects, thus may have translational potential in personalized therapy. The proposed strategy provides a new avenue for the identification of personalized cancer driver mutations in a patient-specific manner.

## MATERIALS AND METHODS

### Homology modeling and mutation analysis

Information about protein domains was based on the Pfam [43] or Interpro [44] database. We used HOPE [45] to analyze the three-dimensional (3D) structural and functional effects of a non-synonymous mutation in the protein. HOPE has been suggested to perform similarly to manual approaches. HOPE collected information from 1) structural calculations on the PDB file base on WHAT IF [46]; if the PDB file was not available, YASARA was used to perform homology modeling, 2) conservation scores estimation by HSSP [47], 3) sequence-based predictions by DAS-servers [48], and 4) sequence annotations by Uniprot. All these data were combined with the known properties of the amino acids in a decision schedule, i.e., the effect of the mutated-allele on the 3D-structure of the protein. We also used the SWISS-model to perform homologous modeling if the crystallographic model was unknown for the interested protein.

### Cell lines

Human hepatoma cell lines (SMMC-7721, SK-Hep1, PLC/PRF/5 and Huh7 cells) and a normal hepatic cell line (LO2) were maintained in Dulbecco's modified Eagle's medium (DMEM; Hyclone^™^, UT). All culture media were supplemented with 10% fetal bovine serum (FBS; Gibco^®^, Rockville MD), 100 units/mL penicillin, and 100 μg/mL streptomycin (Hyclone^™^).

### Plasmids and adenoviruses

Full length coding sequences of human *HNF1A*, *GNMT*, *FAH*, and *IDH1* were amplified by the polymerase chain reaction (PCR) and inserted into the shuttle vector pAdTrack-TO4 (obtained from Dr. T-C He, University of Chicago, Chicago IL). Mutated plasmid of *HNF1A* (S247T), *FAH* (I392V), *IDH1* (V294M), and *GNMT* (A14T) (Table 1) were constructed by overlapping extension PCR and subcloned into pAdTrack-TO4 (primer sequences were listed in Table S1). All recombinant vectors were confirmed by Sanger sequencing. Recombinant wild-type HNF1A (AdHNF1Awt) and S247T-mutated HNF1A (AdHNF1Amut) were generated successfully in HEK293 cells using the AdEasy system. All recombinant adenoviruses expressed green fluorescent protein (GFP) as a marker for monitoring of infection efficiency.

Three pairs of oligonucleotides containing siRNA (Table S2) target sites for the coding region of *HNF1A*, *GNMT*, *FAH*, *IDH1* and *SPTBN1* were designed and subcloned into the Sfi I site of pSES vector (from Dr. T-C He) to generate adenovirus AdR-siHNF1A, AdR-siGNMT, AdR-siFAH, AdR-siIDH1, and AdR-siSPTBN1 using the AdEasy system. A scrambled shRNA control (AdR-siControl) that expresses RFP was used as a control.

The *HNF4A* promoter-luciferase reporter was generated by cloning an approximately 1 kb PCR fragment into the pGL3-Basic vector (Promega, Madison WI; E1751).

### Western blotting

Proteins were extracted from cells with cell lysis buffer (Beyotime Biotechnology, Jiangsu, China) containing 1 mM phenylmethanesulfonyl fluoride (Beyotime). Approximately 50 μg proteins were separated on 10% polyacrylamide gels and electrotransferred to PVDF membranes (Millipore, Billerica MA). The membranes were immunoblotted with the following antibodies: HNF1A (Abcam, Cambridge UK; ab204306), FAH (Bioworld^™^, Atlanta GA; BS8270), IDH1 (Bioworld^™^; BS6918), GNMT (Bioworld^™^; BS8292) and SPTBN1 (Abcam; ab72239). Secondary goat anti-rabbit IgG (H+L)-horseradish peroxidase antibodies were purchased from Bioworld^™^ (BS13278). Endogenous β-actin (Bioworld™; AP0060) expression was used as the normalization control.

### Immunofluorescence staining

Cells were washed twice with phosphate-buffered saline (PBS) and fixed with 4% formaldehyde for 30 min. Cells were then permeabilized with 0.5% Triton and incubated with HNF1A antibody (Abcam; ab204306) at 4^°^C overnight. After washing, cells were incubated with an Alexa Fluor 647-labeled secondary antibody (Invitrogen^®^, Carlsbad CA; A21244) for 1 h at room temperature and counterstained with 4′,6-diamidino-2-phenylindole (DAPI; Roche, Basel, Switzerland) for 5 min. The expression of HNF1A was visualized under a laser scanning confocal microscope (Nikon, Tokyo, Japan; A1+R).

### Cell migration assay

Cell migration was measured using transwell units with a polycarbonate filter (BD, San Jose CA). For knockdown assay, cells were mock-infected or infected with AdR-siHNF1A, AdR-siGNMT, AdR-siFAH, AdR-siIDH1, AdR-siSPTBN1 or AdR-siControl. For overexpression assays, cells were transfected with *HNF1A*-WT, *HNF1A*-Mut (S247T), *GNMT*-WT, *GNMT*-Mut (A14T), *FAH*-WT, *FAH*-Mut (I392V), *IDH1*-WT, *IDH1*-Mut (V294M), or vector control. Twenty-four hours after transduction, cells were suspended in 200 μL of serum-free medium and added at 4 × 10^4^ cells/well in the upper chamber. A DMEM medium (600 μL) containing 10% FBS was added into the lower chamber to act as a chemoattractant. Cells were incubated at 37°C under 5% CO_2_. After 12 h, cells were fixed with 4% formaldehyde and stained with crystal violet. The numbers of migrated cells were counted in five fields (200×) on each membrane and the average per field was calculated.

### Cell proliferation assay

Cell proliferation was measured using the CellTiter 96 AQ One Solution Cell Proliferation Assay (MTS) (Promega). Cells were treated as mentioned in cell migration assay. Infected cells (2 × 10^3)^ were re-plated in 96-well plates, 20 μL of the MTS reagent were added into each well, and the plates were incubated for 2 h at 37°C. The absorbance at 490nm was measured every 24 h until day 4 using a microplate reader (Bio-Tek, Winooski VT).

### Luciferase assay

HepG2 and SMMC-7721 cells were cultured in 25 cm^2^ cell culture flasks and co-transfected with 3 μg of HNF4A responsive luciferase reporter pGL3-HNF4A and pAdTrack-HNF1A wt or pAdTrack-HNF1A mut using Lipofectamine V F2000 (Promega) following the manufacturer's instructions. The pRL-TK plasmid (Promega) was added as an internal control. Cells were harvested 36 h post-transfection and subjected to the Dual-Luciferase^®^ Reporter Assay (Promega). Each assay was performed in triplicate and repeated three times.

### Amplification and sequence analysis of the targeted mutation sites of the five genes

The genome DNA was extracted from hepatoma cell lines using the Wizard Genomic DNA Purification Kit (Promega; A1120) according to the manufacturer's instructions. The targeted mutation sites of the five genes were amplified by PCR and were sequenced by Sanger sequencing. All primer sequences are listed in Table S3.

### Statistical analysis

Pairwise comparison for statistical significance was conducted with Student's *t* test, and analysis of variance (ANOVA) was used to test for the effect of overexpression of the wild- and mutant-type gene in cell proliferation. We considered *p* < 0.05 to be statistically significant.

## SUPPLEMENTARY MATERIALS FIGRES AND TABLES


