# Supplementary material for: Validation of a multi-omics strategy for prioritizing personalized candidate driver genes
Source: Oncotarget. 2016 May 21;7(25):38440–50. doi: 10.18632/oncotarget.9540 (PMC5122402; doi:10.18632/oncotarget.9540)
Supplement: Supplementary file 1 [file oncotarget-07-38440-s001.pdf]

Validation of a multi-omics strategy for prioritizing personalized candidate driver genes

Supplementary Materials

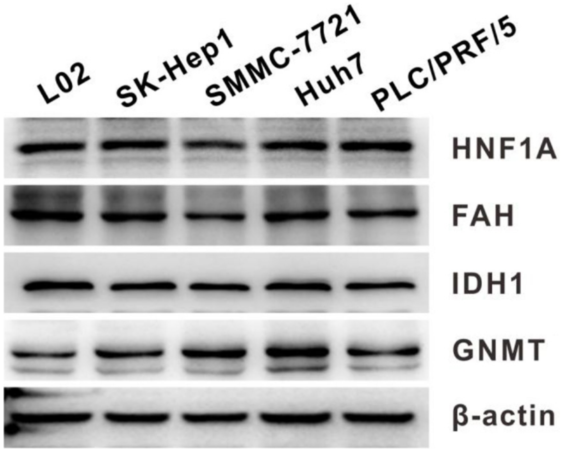

Supplementary Figure S1: The endogenous expression of *HNF1A*, *IDH1*, *GNMT*, and *FAH* in hepatoma cells and normal hepatic cell line L02. Total cell lysates were subjected to immunoblotting with the indicated antibodies. β-actin was used as the loading control.

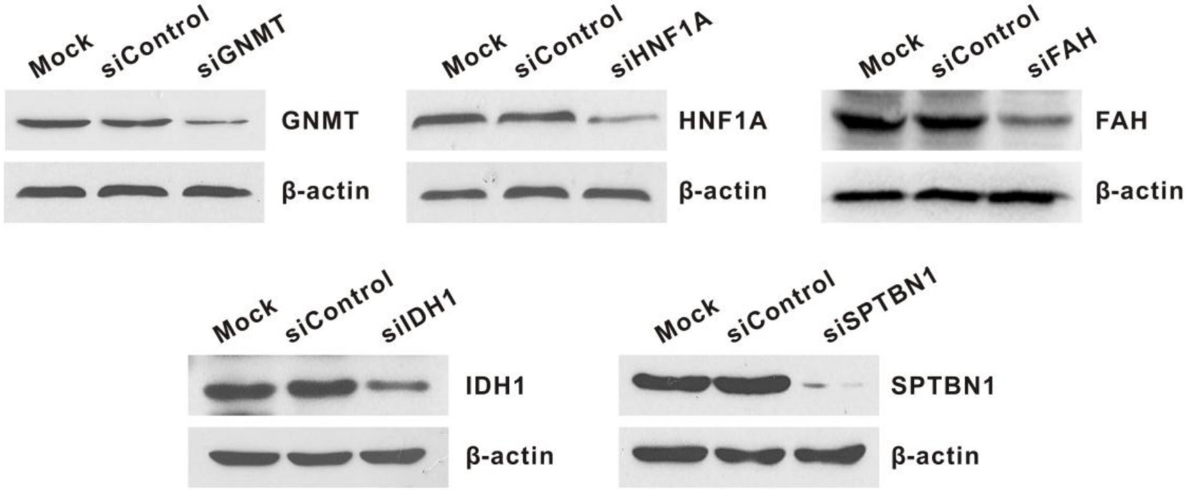

Supplementary Figure S2: Western blot of siRNA for target genes that reduced the endogenous protein expression.

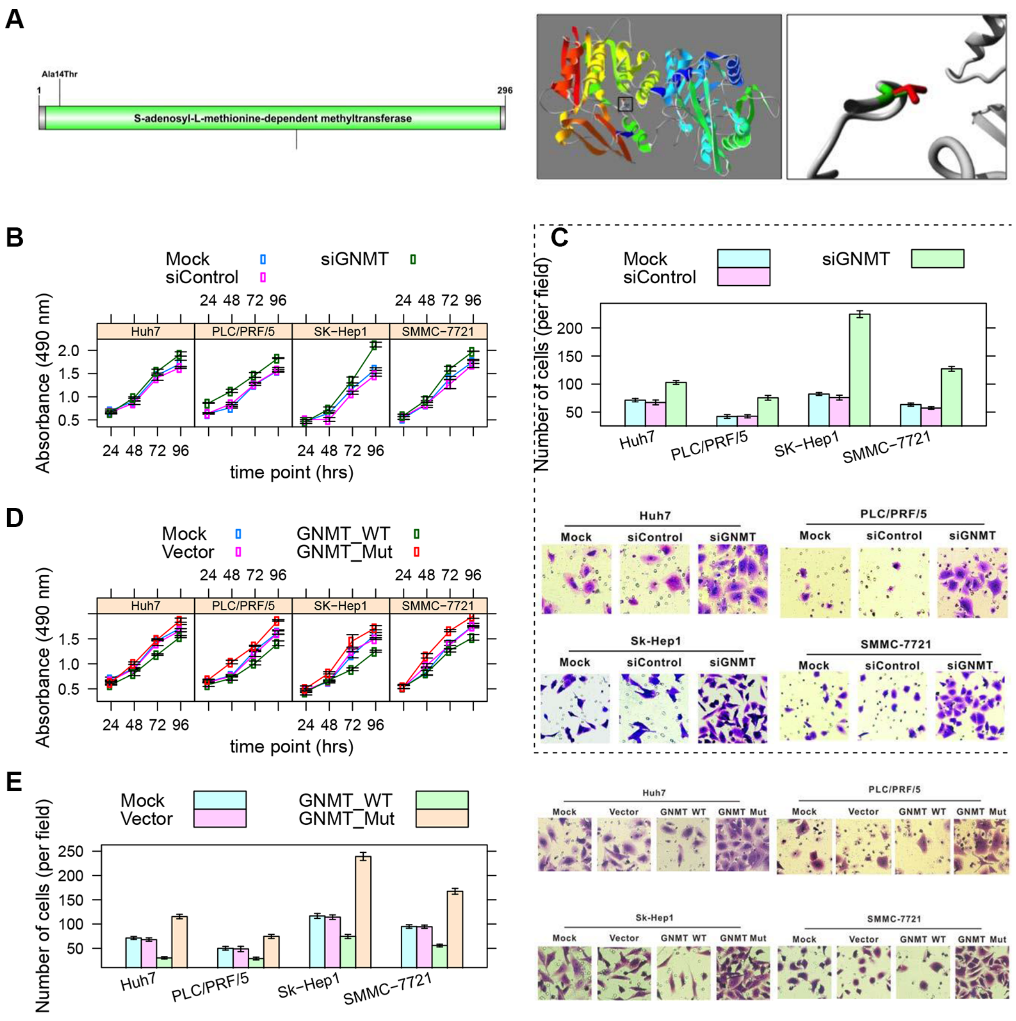

Supplementary Figure S3: Effects of a loss-of-function mutation in *GNMT*. (**A**) Schematic diagram of domains (left) and image of crystallographic model (middle) (PDB: 1R74) of *GNMT*. The Ala14 is located in the SAM-dependent methyltransferase domain. The side chains of both the wild-type and mutant allele residue are shown in green and red, respectively (right). (**B**) Cell growth curve. Hepatoma cell lines SMMC-7721, SK-Hep1, PLC/PRF/5 and Huh7 cells were infected with AdsiGNMT or AdsiControl, respectively. Cell growth was determined by an MTS assay similar to Figure 3B. (**C**) Transwell assay of cell migration property in hepatoma cells depletion of GNMT. Magnification: × 200. (**D**) Hepatoma cells were mock-transfected or transfected with *GNMT*-WT or *GNMT*-Mut (A14T), respectively. Tumor cell growth was measured by a MTS assay. (**E**) Cells were treated as described in (D). Cell migration was determined by a transwell assay.

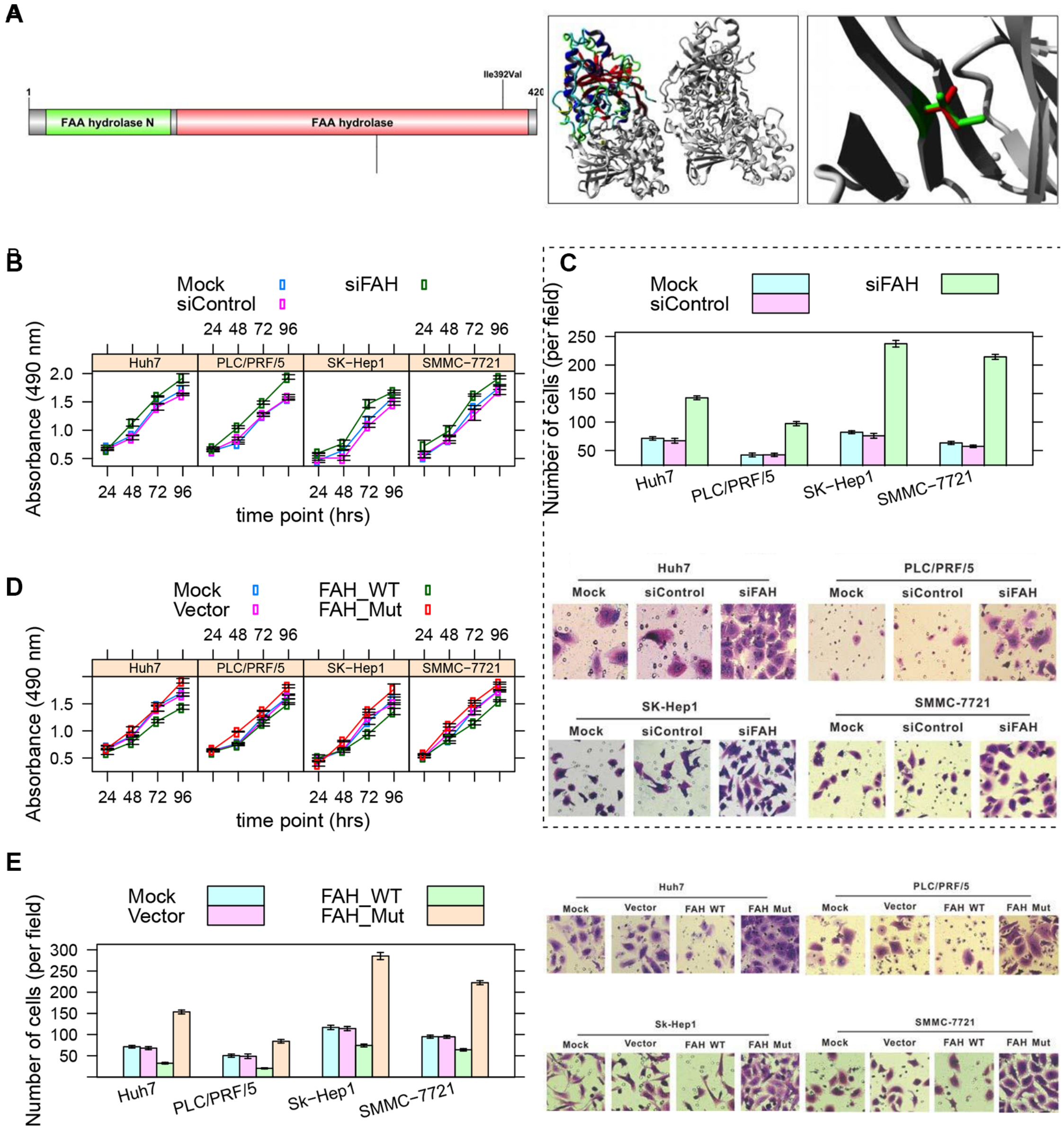

Supplementary Figure S4: Effects of a loss-of-function mutation in *FAH*. (**A**) Schematic diagram of domains (left) and image of crystallographic model based on homologous modeling (right) upon mouse Fah (PDB: 1HYO). The I392 residue is located in the FAA hydrolase domain. The side chains of both the wild-type and mutant allele residue are shown in green and red, respectively (right). (**B**) Cell growth curve. Hepatoma cell lines SMMC-7721, SK-Hep1, PLC/PRF/5 and Huh7 cells were infected with AdsiFAH or AdsiControl, respectively. Cell growth was determined by MTS assay similar to Figure 3B. (**C**) Transwell assay of cell migration property in hepatoma cells depletion of *FAH*. Magnification: × 200. (**D**) Hepatoma cells were mock-transfected or transfected with *FAH*-WT or *FAH*-Mut (I392V) respectively. Tumor cell growth was measured by MTS assay. (**E**) Cells were treated as describe in (D). Cell migration were determined by a transwell assay.

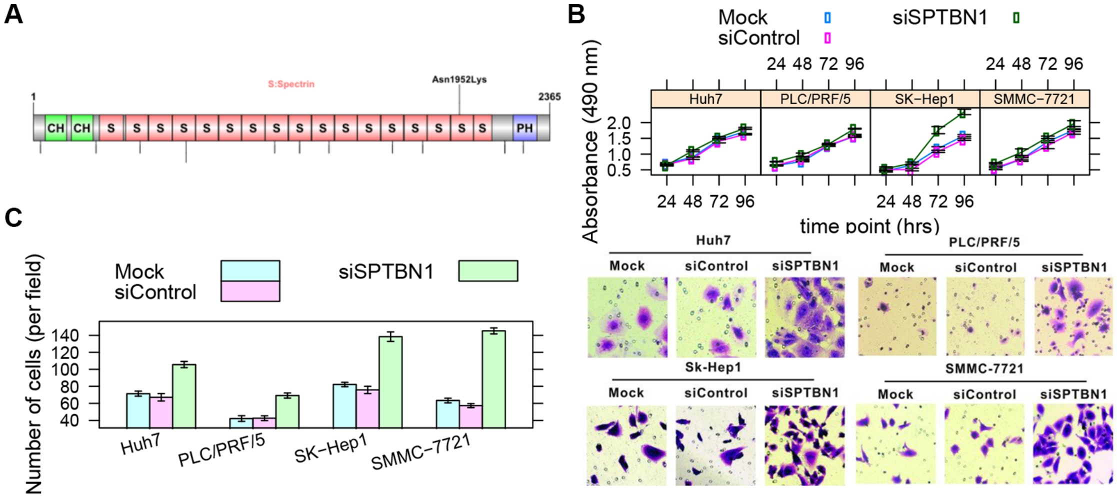

Supplementary Figure S5: A mutation in *SPTBN1*. (**A**) Schematic diagram of domains in *SPTBN1*, and the N1952 residue is located in one of the spectrin repeats (S). (**B**) Cell growth curve. Hepatoma cell lines SMMC-7721, SK-Hep1, PLC/PRF/5 and Huh7 cells were infected with AdsiSPTBN1 or AdsiControl, respectively. Cell growth was determined by an MTS assay similar to Figure 3B. (**C**) A transwell assay of cell migration property in hepatoma cells depletion of *SPTBN1*. Magnification: × 200.

Supplementary Table S1: Primers for coding regions of *HNF1A*, *FAH*, *IDH1*, *GNMT*, and *SPTBN1*, and their mutants

PCR product	Sense primer (5′–>3′)	Antisense primer (5′–>3′)
HNF1A	catggatccaccatggtttctaaactgagccagctgcag	tacaagcttttactgggaggaagaggccatctgggt
HNF1A S247T	atccagagaggggtgaccccatcacaggcacag	tacaagcttttactgggaggaagaggccatctgggt
FAH	catggatccaccatgggctccttcatcccggtggccgagga	tacaagctttcatgatggcaggagagcaggcagcac
FAH I392V	ggggatgaagtcatcgtaacagggtactgccag	tacaagctttcatgatggcaggagagcaggcagcac
IDH1	catggatccaccatgggctccaaaaaaatcagtggcggtt	tactctagattaaagtttggcctgagctagttt
IDH1 V294M	ggcatgatgaccagcatgctggtttgtccagat	tactctagattaaagtttggcctgagctagttt
GNMT	catggatccaccatggtggacagcgtgtaccggacc	tacaagctttcagtctgtcctcttgagcacgtggat
GNMT A14T	tccctgggggtgacggccgaagggct	tacaagctttcagtctgtcctcttgagcacgtggat

Supplementary Table S2: siRNA primers

PCR product	Sense primer (5′–>3′)	Antisense primer (5′–>3′)
siHNF1A 1	atcaaagagctggagaaccttttt	aaggttctccagctctttgatttt
siHNF1A 2	acgaagatggtcaagtcctatttt	ataggacttgaccatcttcgtttt
siHNF1A 3	agcaaagaggcactgatccatttt	atggatcagtgcctctttgctttt
siFAH 1	aatgttcagggacaaggagatttt	atctccttgtccctgaacattttt
siFAH 2	aggctttggccagtgtgctgtttt	acagcacactggccaaagcctttt
siIDH1 1	aggagaaaacttgaagatcatttt	atgatcttcaagttttctcctttt
siIDH1 2	acaaactagctcaggccaaatttt	atttggcctgagctagtttgtttt
siIDH1 3	atcaaactagctcaggccaatttt	attggcctgagctagtttgatttt
siGNMT 1	accagtgacaagatgctgaatttt	attcagcatcttgtcactggtttt
siGNMT 2	atgtggatgccagtgacaagtttt	atcagcatcttgtcactggctttt
siGNMT 3	atgtggatgccagtgacaagtttt	acttgtcactggcatccacatttt
siSPTBN1 1	aagaccttgctgaagagagatttt	atctctcttcagcaaggtcttttt
siSPTBN1 2	aaagacaacaaagagaagaatttt	attcttctctttgttgtcttttttt
siSPTBN1 3	acaacaaggcctgggaaagatttt	atctttcccaggccttgttgtttt
pGL3-HNF4A	tacggtaccgctcaggaaggcaatgtgagacctgtt	cataagcttcatctaacatatttattgagcaccta

Supplementary Table S3: DNA sequencing primers

PCR product	Sense primer (5′–>3′)	Antisense primer (5′–>3′)
HNF1A	cctggcattggaacccagat	cccttgtccccacataccac
GNMT	gatggtggacagcgtgtacc	gttccaggattggcaaaggc
IDH1	agactcagtgctcttcatgca	tggtgatgactttgcacaca
FAH	tctgcagtgatcccaccaag	cagagactctgggaggcaga
SPTBN1	gctcagggagtgattcagacc	tctagacgcacaggaaagga
